# Telitacicept as an alternative to non-steroidal immunosuppressive therapies in the treatment of myasthenia gravis: a study on clinical efficacy and steroid-sparing effect

**DOI:** 10.3389/fimmu.2025.1549034

**Published:** 2025-03-24

**Authors:** Zheyu Fang, Yuan Zhang, Yu Zhang, Qiaoyi Zhang, Xi Qu, Shengli Pan, Bingbing Wan, Shiyin Yang, Xu Zhang, Jia Li

**Affiliations:** Department of Neurology, The First Affiliated Hospital of Wenzhou Medical University, Wenzhou, Zhejiang, China

**Keywords:** myasthenia gravis, telitacicept, minimal symptom expression, steroid-sparing, non-steroidal immunosuppressive therapies

## Abstract

**Introduction:**

Myasthenia Gravis (MG) is an autoimmune disorder characterized by impaired neuromuscular junction (NMJ) transmission. Current treatments for MG include steroids and nonsteroidal immunosuppressive therapies (NSISTs). However, approximately 20% of patients show a poor response to these therapies, which are often associated with significant side effects. Telitacicept, a novel recombinant fusion protein targeting the BAFF/APRIL pathway, has shown promise in treating autoimmune diseases, including MG.

**Methods:**

This retrospective study compared the efficacy of telitacicept monotherapy (10 patients) to NSISTs (16 patients) and sequential therapy (6 patients) in managing Myasthenia Gravis (MG) at The First Affiliated Hospital of Wenzhou Medical University (July 2020-November 2024). The primary endpoint was the time to achieve minimal symptom expression (MSE), and secondary endpoint was the change in the mean daily prednisone dosage from baseline to month 4.

**Results:**

Among telitacicept-treated patients, 80% achieved MSE within 4 months, with a significant reduction in mean daily dose of prednisone (from 45.00 mg to 6.25 mg, *P* < 0.001). In contrast, only 12.5% of the NSISTs group achieved MSE, with no significant change in mean daily dose of prednisone (*P* = 0.091). The sequential therapy group (efgartigimod followed by telitacicept) maintained stable disease conditions.

**Conclusion:**

Telitacicept is effective in inducing MSE rapidly and offers a steroid-sparing effect, making it a promising alternative to traditional NSISTs with fewer side effects in MG patients.

## 1 Introduction

Myasthenia Gravis (MG) is an autoimmune disorder primarily mediated by acetylcholine receptor (AChR) antibodies, characterized by T-cell dependent mechanisms and complement involvement, resulting in impaired neuromuscular junction (NMJ) transmission. Clinically, MG presents with symptoms such as ptosis, diplopia, and difficulties in facial expressions, speech, and swallowing. The disease typically begins with ocular muscle involvement and may progress to generalized muscle weakness, affecting the proximal muscles of the limbs and trunk (1–3). Autoantibodies targeting the postsynaptic AChR at the NMJ are the main pathogenic antibodies. Additionally, antibodies against muscle-specific tyrosine kinase (MuSK), low-density lipoprotein receptor-related protein 4 (LRP4), and ryanodine receptor (RyR) contribute to MG pathogenesis by disrupting AChR clustering, affecting AChR function, and NMJ signaling (4). In recent years, minimal symptom expression (MSE), defined as an MG activities of daily living (MG-ADL) score of ≤ 1, has been used as a metric to evaluate therapeutic goals in MG (5–10).

Current MG treatments include acetylcholinesterase inhibitors, conventional immunosuppressants (such as corticosteroids, non-steroidal immunosuppressants [NSISTs] like tacrolimus, azathioprine, and mycophenolate mofetil), plasma exchange (PLEX), and intravenous immunoglobulin (IVIG). For about 10-15% of MG patients with complications such as thymoma and stable disease, thymectomy is considered (11). While these therapeutic approaches effectively control the condition in most patients, about 20% of patients show a poor response to conventional immunosuppressive therapy. Additionally, steroids and NSISTs often have significant side effects, such as diabetes, osteoporosis, hypertension, and obesity. As a result, long-term adherence to treatment can be challenging for some patients (12–15). Therefore, researchers have been developing new drugs with more targeted action, higher safety, and better efficacy in recent years (16).

The pathogenic role of B cells in MG includes the production of pathogenic autoantibodies and the regulation of immune responses through cytokine and chemokine production. B-cell stimulating factor (BLyS, also known as B-cell activating factor, BAFF) and APRIL (a proliferation-inducing ligand) are key factors in maintaining the B-cell pool and humoral immunity. Specifically, BLyS regulates the differentiation and maturation of immature B cells, while APRIL controls the function and survival of long-lived plasma cells. These factors play an important role in the pathogenesis of autoimmune diseases. Targeting the BAFF/APRIL pathway has been proposed as a mechanistic approach for treating generalized MG (gMG) and other autoimmune diseases (12).

Telitacicept is a novel recombinant fusion protein composed of the ligand-binding domain of the transmembrane activator and calcium modulator and cyclophilin ligand interactor (TACI) receptor and the Fc component of human IgG. It binds to and neutralizes the activity of BAFF and APRIL, thereby inhibiting the maturation and differentiation of B cells and plasma cells at multiple stages (17, 18). These mechanisms are crucial in the pathogenesis of various autoimmune diseases. Telitacicept was approved for the treatment of systemic lupus erythematosus (SLE) in China in March 2021, and has shown promise in the treatment of IgG4-related disease, rheumatoid arthritis, and neuromyelitis optica spectrum disorders (17, 19). In phase II clinical trials for MG, telitacicept significantly reduced the clinical severity of gMG and demonstrated good safety (12). Ongoing phase III trials have shown promising results, with telitacicept continuing to improve clinical outcomes in gMG patients, while maintaining favorable safety profiles (data not yet published). Several case reports also highlight the therapeutic potential of telitacicept in MG (18, 20, 21).

However, to date, telitacicept has primarily been used as an adjunctive therapy rather than as a replacement for NSISTs. Despite the promising potential of these new targeted therapies, their role remains supplementary rather than as independent alternatives to NSISTs. Data on their use as primary treatment options for MG is still limited. Therefore, there is a significant need for research into innovative, effective alternative therapies that can provide stronger disease control with fewer side effects compared to traditional treatments.

Given the proven efficacy of telitacicept in a range of autoimmune diseases, including MG, there has been no investigation into whether it can replace NSISTs. This study aims to evaluate the clinical efficacy of telitacicept as an alternative therapy to NSISTs in the treatment of MG and its potential for steroid-sparing effects. Through this research, we hope to provide MG patients with a new treatment option that can rapidly achieve MSE while minimizing the side effects associated with long-term steroid use.

## 2 Materials and methods

### 2.1 Study design and patients

This was a retrospective, single-center study conducted at the Department of Neurology, The First Affiliated Hospital of Wenzhou Medical University, from July 2020 to November 2024. The study aimed to evaluate the clinical efficacy and steroid-sparing effect of telitacicept in patients with MG. The analysis included two retrospective cohorts (1): 10 patients treated with telitacicept monotherapy, and (2) 6 patients receiving a sequential treatment of efgartigimod and telitacicept. Additionally, a control group of 16 patients treated with traditional NSISTs was included for comparison with the first cohort.

Patients eligible for inclusion were diagnosed with MG according to the 2020 Chinese Guidelines for the Diagnosis and Treatment of MG. Diagnosis was confirmed based on clinical features, particularly fluctuating and fatigable muscle weakness, and supported by at least one positive result from pharmacological testing, serum antibody measurement, or a positive result from repetitive nerve stimulation (RNS) testing. All patients had complete medication histories. The Myasthenia Gravis Foundation of America (MGFA) classification scale was used to assess disease severity.

In this study, instances where patients were unable to tolerate the toxicity of immunosuppressive therapy included adverse reactions such as severe osteoporosis, avascular necrosis of the femoral head, renal insufficiency, and diabetes, as well as contraindications related to pre-existing comorbidities. The exclusion criteria were as follows: severe acute infections prior to the initiation of treatment, pregnant or lactating women, patients with known allergies to biologics, and individuals with significant hepatic dysfunction.

### 2.2 Endpoints

Primary Efficacy Endpoint: The primary efficacy endpoint was the time to achieve MSE, defined as the absence or only mild MG symptoms, with an MG-ADL score ≤ 1. Clinical assessments were performed at baseline, 2 months, and 4 months. For patients in the sequential treatment group, MG-ADL scores were collected at weeks 4, 5, 6, 9, 13, 17, and 21. Secondary Efficacy Endpoints: The secondary efficacy endpoint was the change in the mean daily prednisone dosage from baseline to month 4. The daily dose of prednisone was recorded for each patient group at baseline, 2 months, and 4 months. For the sequential cohort, prednisone doses were collected at weeks 4, 8, 12, and 16.

### 2.3 Dosing regimen

An individualized dosing regimen for the telitacicept group was employed, with adjustments made based on the severity of disease manifestations and economic considerations. Initially, patients received either 240 mg once weekly (qw) or 160 mg qw for the first four weeks. Following clinical evaluation, the dosing interval was progressively extended, and concurrent corticosteroid use was gradually reduced. Within one month, five patients had their dosing interval extended to once every two weeks (q2w), while three patients achieved a q2w interval within two months.

The sequential therapy protocol was structured in two phases. Phase 1 involved the administration of four efgartigimod injections at 10 mg/kg over three weeks (weeks 0, 1, 2, and 3). Phase 2 introduced telitacicept therapy starting at week 4, with a 240 mg dose per injection. Following the achievement of clinical stability, the interval between telitacicept doses was progressively lengthened, with subsequent injections administered at weeks 4, 5, 6, 7, 9, 11, 13, 17, and 21.

The dosing schedule was tailored to several factors, including the pharmacokinetic properties of the drug (19, 22), previous research on dosing intervals, and cost-effectiveness considerations. Notably, the inclusion of the IgG Fc fragment in telitacicept extends its half-life in circulation, allowing for a more flexible dosing schedule. Due to the high treatment costs, the dosing frequency was initially adjusted from weekly to bi-weekly, and ultimately to a monthly regimen.

This personalized approach aimed to balance therapeutic efficacy with economic feasibility, optimizing treatment frequency while maintaining clinical stability. By extending dosing intervals, the strategy sought to minimize the treatment burden on patients, considering both the financial and adherence-related challenges associated with prolonged therapy.

### 2.4 Safety evaluation

The safety of the treatments was assessed by monitoring the occurrence of adverse events (AEs), including both common and serious adverse events. All adverse events were classified according to the Common Terminology Criteria for Adverse Events, and the severity of each AE was recorded.

### 2.5 Statistical analysis

Descriptive statistics were used to summarize baseline characteristics and clinical outcomes. Continuous variables were presented as mean ± standard deviation (SD) or median (interquartile range, IQR), depending on their distribution. Categorical variables were expressed as absolute numbers and percentages (%). Normal distribution was assessed using the Shapiro-Wilk test. For continuous variables, comparisons between groups were made using either the Mann-Whitney U test for non-normally distributed data or the independent samples t-test for normally distributed data. Longitudinal analysis across multiple time points was conducted with one-way repeated measures ANOVA for normally distributed datasets and the Friedman test for non-parametric variables. Additionally, Mann-Kendall trend analyses were conducted to assess the changes in trends of MG-ADL scores. All statistical analyses were conducted using SPSS software (version 27.0.1) or R software (Version 4.2.1), with graphical representations created using Prism (Version 8.0.1). A *P*-value < 0.05 was considered statistically significant.

## 3 Result

### 3.1 Clinical outcomes and steroid-sparing effects of telitacicept in MG patients

In this retrospective analysis of MG, we included 10 patients (4 males, 6 females). The mean age was 49.90 ± 23.59 years, ranging from 17 to 81 years, and the median disease duration prior to treatment was 37.00 months (IQR 4.75-182.00 months). The majority of patients (4 cases, 40.0%) were classified as MGFA Class I, while the remaining 6 patients (60.0%) were distributed across Class II to V. Serological testing revealed that two patients (20.0%) were positive for AChR antibodies, two patients (20.0%) were antibody-negative, and the remaining six patients (60.0%) did not undergo antibody testing. One patient (10.0%) was diagnosed with thymoma, and two patients (20.0%) had thymic hyperplasia, all of whom underwent thymectomy. At baseline, nine patients (90%) had a daily steroid dose of ≥ 30 mg (**Table 1**).

**Table 1:** Baseline characteristics of enrolled patients treated with telitacicept monotherapy.

Patient No.	1	2	3	4	5	6	7	8	9	10
Age (years)	42	40	70	75	17	35	78	31	81	30
Gender	Male	Female	Male	Male	Female	Female	Female	Female	Male	Female
Disease duration (months)	392	157	50	4	1	24	257	12	5	108
MGFA class	IIa	I	I	I	IIa	I	IVa	IIIb	IIIb	IIIa
Thymus status	Thymic hyperplasia	Normal	Thymoma	Normal	Thymic atrophy	Thymic hyperplasia	Normal	Normal	Normal	Normal
Thymectomy	Y	N	Y	N	N	Y	N	N	N	N
Duration of thymectomy (months)	157	N	30	N	N	4	N	N	N	N
Ab subtypes	NA	NA	NA	NA	Negative	NA	Negative	NA	AChR-Ab (+)	AChR-Ab (+)
Ab concentration (nmol/L or titre)	NA	NA	NA	NA	N	NA	N	NA	19.302	> 20.00
RNS	NA	NA	NA	Positive	Negative	Negative	NA	NA	NA	NA
Fatigue test	Positive	Positive	Positive	Positive	Positive	Positive	Positive	Positive	Positive	Positive
Baseline MG-ADL Score^a^	3	4	3	3	3	3	6	7	6	9
Comorbidities	Hyperthyroidism	N	HTN, Left lung malignant tumor, right lung cryptococcosis	Gastrointestinal bleeding	N	Thalassemia	HTN, DM	N	HTN, DM, Syphilis, COPD, Hepatic insufficiency	Fatty liver, Hyperuricemia
Previous MG medications	Pyridostigmine bromide, Prednisone, MMF, TAC	Pyridostigmine bromide, Prednisone, TAC	Pyridostigmine bromide, Prednisone, MMF	Prednisone, TAC	Pyridostigmine bromide, Prednisone	Pyridostigmine bromide, Prednisone	Pyridostigmine bromide, Prednisone, MMF, TAC	Pyridostigmine bromide, Prednisone	Pyridostigmine bromide, Prednisone, MMF, TAC	Pyridostigmine bromide, Prednisone
Initial dose of GCS (mg/d)	90	60	60	30	30	90	0	30	60	30

MGFA, Myasthenia Gravis Foundation of America; AChR-Ab, acetylcholine receptor antibodies; RNS, repetitive nerve stimulation; HTN, hypertension; DM, diabetes mellitus; COPD, Chronic obstructive pulmonary disease; MG-ADL, Myasthenia Gravis–Activities of Daily Living; Ab, antibody; MMF, mycophenolate mofetil; TAC, tacrolimus; GCS, Glucocorticoids; NA, not available.

a Total MG -ADL scores range from 0 (normal) to 24 (severe).

**Figure 1:**
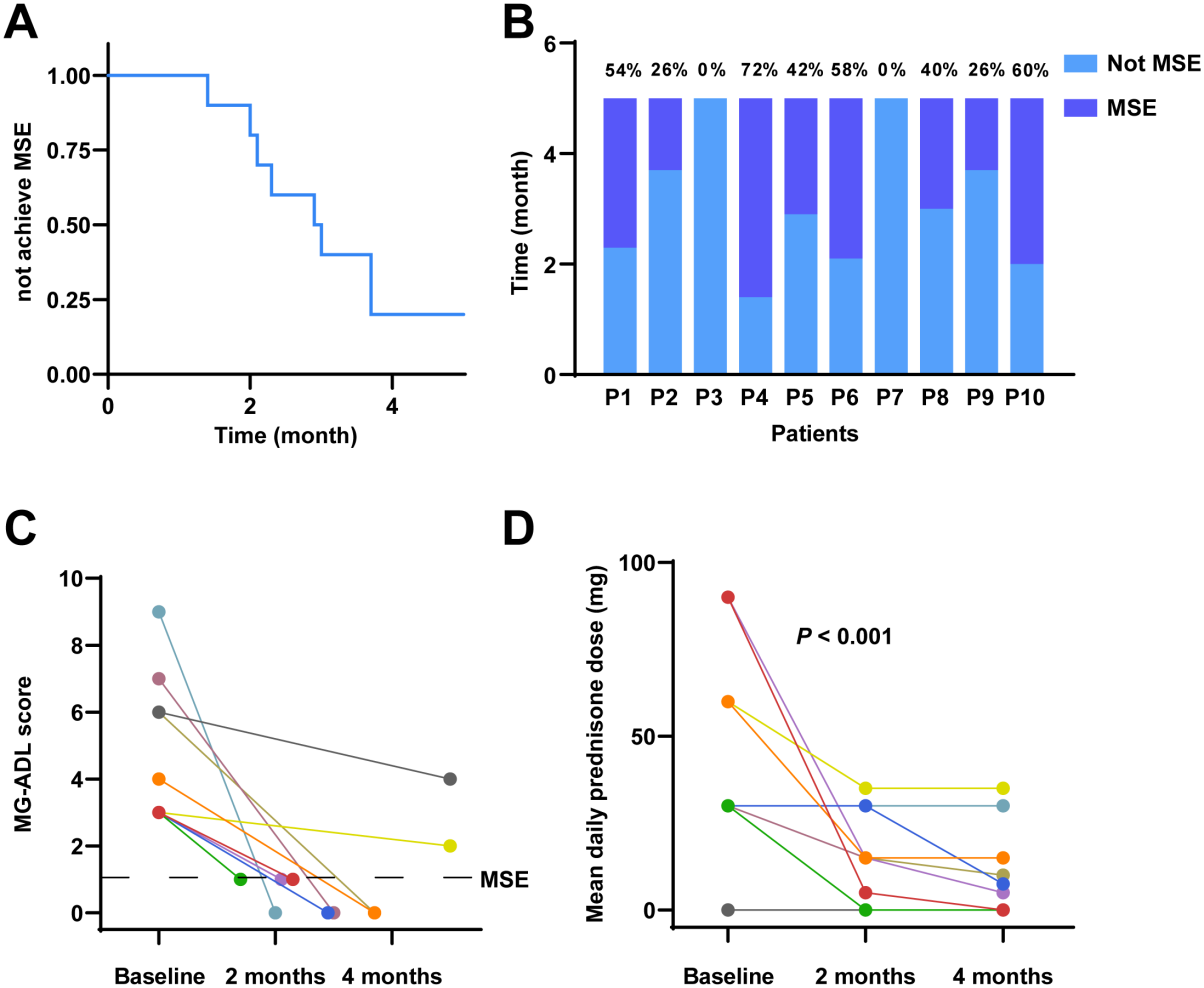
Clinical outcomes following telitacicept treatment in MG. **(A)** Kaplan-Meier survival curve illustrating the probability of not achieving MSE over a 5-month period. **(B)** The proportion of the 5-month period during which each patient was in MSE. **(C)** Individual MG-ADL scores and time to MSE. **(D)** Mean daily prednisone dose at baseline, 2 months, and 4 months.

All patients completed a 5-month clinical follow-up. During this period, 80% of patients (8 out of 10) achieved the MSE. Specifically, two patients reached MSE within 2 months, four patients within 3 months, and two patients within 4 months. The percentage of time spent in MSE status over the 5-month study period was as follows: 54%, 26%, 72%, 42%, 58%, 40%, 26% and 60% (**Figures 1A–C**).

The mean daily prednisone dosage at baseline, 2 months, and 4 months is illustrated in **Figure 1D**. Accordingly, the mean daily dose of prednisone decreased from 45.00 mg (IQR 30.00-67.50 mg) at baseline to 15.00 mg (IQR 3.75-30.00 mg) at 2 months, and further reduced to a median of 6.25 mg (IQR 0-18.75 mg) at 4 months. Notably, among the patients who did not achieve MSE during the study period were Patient 3 and Patient 7. For Patient 3, the mean daily dose of prednisone decreased from 60.00 mg at baseline to 35.00 mg at 2 months and remained at 35.00 mg at 4 months. Patient 7 did not receive any prednisone during the study period. Friedman’s test demonstrated a significant reduction in the mean daily prednisone dosage over the study period (*P* < 0.001), indicating a statistically significant steroid-sparing effect.

There were no instances of treatment discontinuation or interruption due to death, serious adverse drug reactions (SADR), or serious treatment-emergent adverse events (SAE). One patient (10.0%) experienced transient pain and swelling at the injection site, and the symptoms resolved spontaneously within a few days. Additionally, there was a single instance of a mild upper respiratory infection that was effectively managed and resolved within a week.

### 3.2 Stable disease conditions in patients treated with sequential therapy of efgartigimod and telitacicept

In this second retrospective analysis, we included 6 patients (1 male, 5 females). The mean age at disease onset was 61.67 ± 10.76 years, ranging from 46 to 78 years, and the mean disease duration prior to treatment was 36.17 ± 41.98 months. The majority of patients (3 cases, 50.0%) were classified as MGFA Class IIIa, with two patients (accounting for 33.3%) classified as Class IIIb, and one case as Class V. Serological testing revealed AChR antibody positivity in 4 patients (66.7%), and another patient positive for MuSK antibodies (16.7%). One patient was diagnosed with thymoma and underwent thymectomy. At baseline, all patients had a daily steroid dose of ≥ 30 mg (**Table 2**).

**Table 2:** Baseline characteristics of enrolled patients receiving a sequential treatment of efgartigimod and telitacicept.

Patient No.	1	2	3	4	5	6
Age (years)	58	62	58	78	68	46
Gender	Female	Male	Female	Female	Female	Female
Disease duration (months)	54	7	108	2	1	45
MGFA class	IIIa	IIIa	IIIb	IVa	IIIa	IIIb
Duration of thymectomy (months)	N	N	N	N	N	46
Ab subtypes	AChR-Ab + Tintin-Ab	AChR-Ab + Tintin-Ab + Ryr-Ab	AChR-Ab	MuSK-Ab	Negative	AChR-Ab
RNS	Positive	NA	NA	Negative	Negative	NA
Fatigue test	Positive	Positive	Positive	Positive	Positive	Positive
Thymus status	Normal	Normal	Thymic hyperplasia	Normal	Normal	Thymoma
Thymectomy	N	N	N	N	N	Yes
Baseline MG-ADL Score^b^	3	5	7	6	6	7
Baseline QMG Score^a^	14	11	6	10	13	15
Comorbidities	N	HTN, OP, Bone fracture, Spinal Infection, CHB	N	HTN, CHD with stent history	CHB, HTN	N
Pre-protocol MG medications	N	Pyridostigmine bromide, Prednisone	Pyridostigmine bromide	N	Pyridostigmine bromide, Prednisone	Pyridostigmine bromide, Prednisone
Initial GCS dose post-protocol (mg/d)	60	30	60	60	60	30

MGFA, Myasthenia Gravis Foundation of America; Ab, antibody; AChR-Ab, acetylcholine receptor antibodies; MuSK-Ab, muscle specific tyrosine kinase antibodies; RNS, repetitive nerve stimulation; MG-ADL, Myasthenia Gravis–Activities of Daily Living; QMG, quantitative myasthenia gravis; HTN, hypertension; OP, Osteoporosis; CHB, Chronic Hepatitis B; CHD, Coronary Heart Disease; GCS, Glucocorticoids; NA, not available.

a total QMG scores range from 0 (none) to 39 (severe).

b Total MG-ADL scores range from 0 (normal) to 24 (severe).

**Figure 2:**
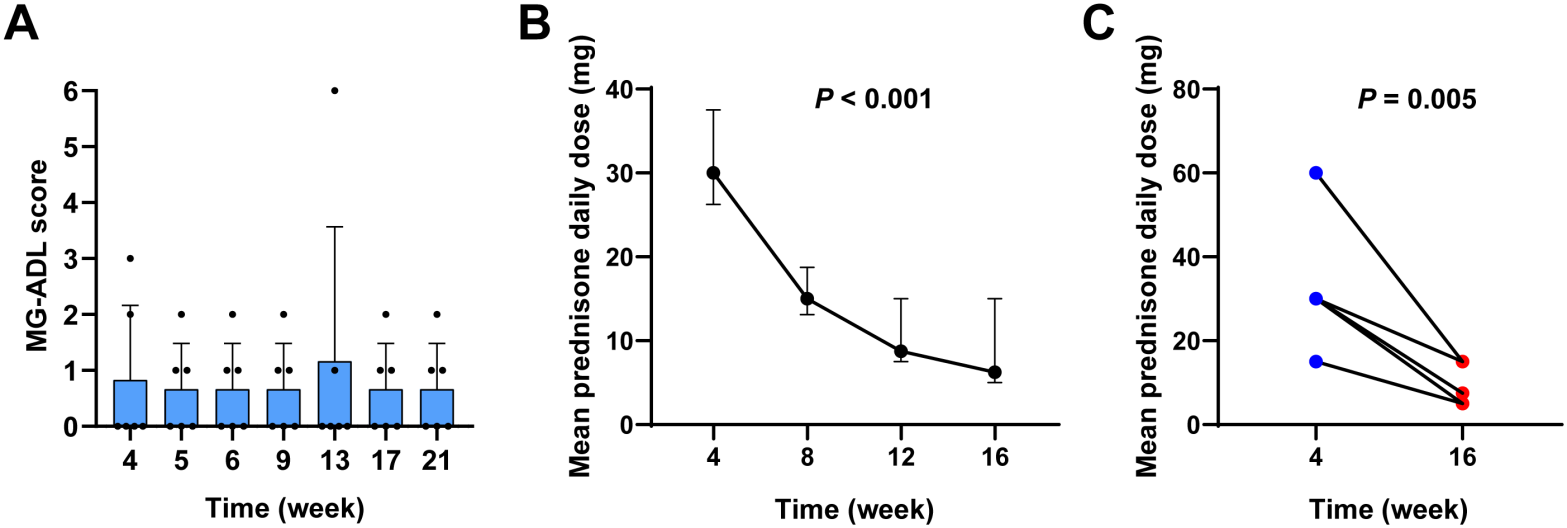
Stability of MG in patients treated with sequential therapy of efgartigimod and telitacicept. **(A)** MG-ADL scores at weeks 4, 5, 6, 7, 9, 11, 13, 17, and 21. **(B)** A reduction in mean daily prednisone dosage was observed in patients undergoing sequential therapy at the 16-week follow-up. **(C)** Comparison of mean daily prednisone dose at week 4 and week 16.

**Figure 2A** shows the MG-ADL scores over time for all patients. The MK trend test yielded a *Z*-value of -0.38023 and a *P*-value of 0.7038, suggesting no significant upward trend in MG-ADL scores over time. Additionally, Friedman’s test confirmed that there were no statistically significant differences in MG-ADL scores at different time points (*P* > 0.05), indicating that the disease did not significantly worsen or progress during the telitacicept treatment period.

Accordingly, in this cohort, following sequential treatment with telitacicept, the mean daily dose of prednisone decreased from 30.00 mg (IQR 26.25-37.50 mg) at 4 weeks to 15.00 mg (IQR 13.125-18.75 mg) at 8 weeks, and further reduced to a median of 8.75 mg (IQR 7.50-15.00 mg) at 12 weeks, and finally reduced to a median of 6.25 mg (IQR 5.00-15.00 mg) at 16 weeks (**Figure 2B**). Friedman’s test demonstrated a significant reduction in the mean daily prednisone dosage over the study period (*P* < 0.001). A comparison between the 4-week and 16-week time points revealed that the average daily prednisone dose at 16 weeks was significantly lower than at 4 weeks (*P* = 0.005), indicating a statistically significant steroid-sparing effect (**Figure 2C**).

### 3.3 Rapid achievement of MSE and steroid-sparing effects with telitacicept compared to NSISTs therapies

To further investigate whether telitacicept could replace NSISTs, we included 16 additional patients who were treated exclusively with conventional immunosuppressive agents as a control group. The baseline characteristics of these patients are detailed in **Supplementary Table 1**. **Table 3** compares the baseline characteristics of the telitacicept group and the NSISTs group. There were no significant differences between the two groups in terms of gender, age, disease duration, MGFA classification, thymic status, antibody status, RNS, initial ADL score, or initial steroid dosage (all *P* > 0.05) (**Supplementary Table 1**).

**Table 3:** Baseline characteristics of enrolled patients.

Characteristics	NSISTs (n = 16)	Telitacicept (n = 10)	*P*-value
Gender, n (%)			0.16
Female	14 (87.5%)	6 (60%)	
Male	2 (12.5%)	4 (40%)	
Age, mean ± SD	49.25 ± 15.21	49.90 ± 23.59	0.94
Disease duration (months), median (IQR)	12.00 (3.25, 26.25)	37.00 (4.75, 182.00)	0.15
MGFA class, n (%)			0.37
OMG	3 (18.8%)	4 (40%)	
GMG	13 (81.2%)	6 (60%)	
Thymus status, n (%)			0.49
Normal	10 (62.5%)	6 (60%)	
Thymoma	4 (25%)	1 (10%)	
Thymic hyperplasia	2 (12.5%)	2 (20%)	
Thymic atrophy	0 (0%)	1 (10%)	
Ab status, n (%)			0.21
AChR-Ab	8 (72.7%)	2 (50%)	
MuSK-Ab	2 (18.2%)	0 (0%)	
Negative	1 (9.1%)	2 (50%)	
RNS, n (%)			0.52
Positive	9 (69.2%)	1 (33.3%)	
Negative	4 (30.8%)	2 (66.7%)	
Baseline MG-ADL Score^a^, mean ± SD	6.56 ± 2.28	4.90 ± 2.02	0.07
Initial dose of GCS (mg/d), mean ± SD	41.25 ± 16.18	48.00 ± 28.98	0.51

MGFA, Myasthenia Gravis Foundation of America; OMG, Ocular Myasthenia Gravis; GMG, Generalized Myasthenia Gravis; SD, Standard Deviation; IQR, Interquartile Range; AChR-Ab, acetylcholine receptor antibodies; MuSK-Ab, muscle specific tyrosine kinase antibodies; RNS, repetitive nerve stimulation; MG-ADL, Myasthenia Gravis–Activities of Daily Living; Ab, antibody; GCS, Glucocorticoids.

a Total MG -ADL scores range from 0 (normal) to 24 (severe).

**Figure 3:**
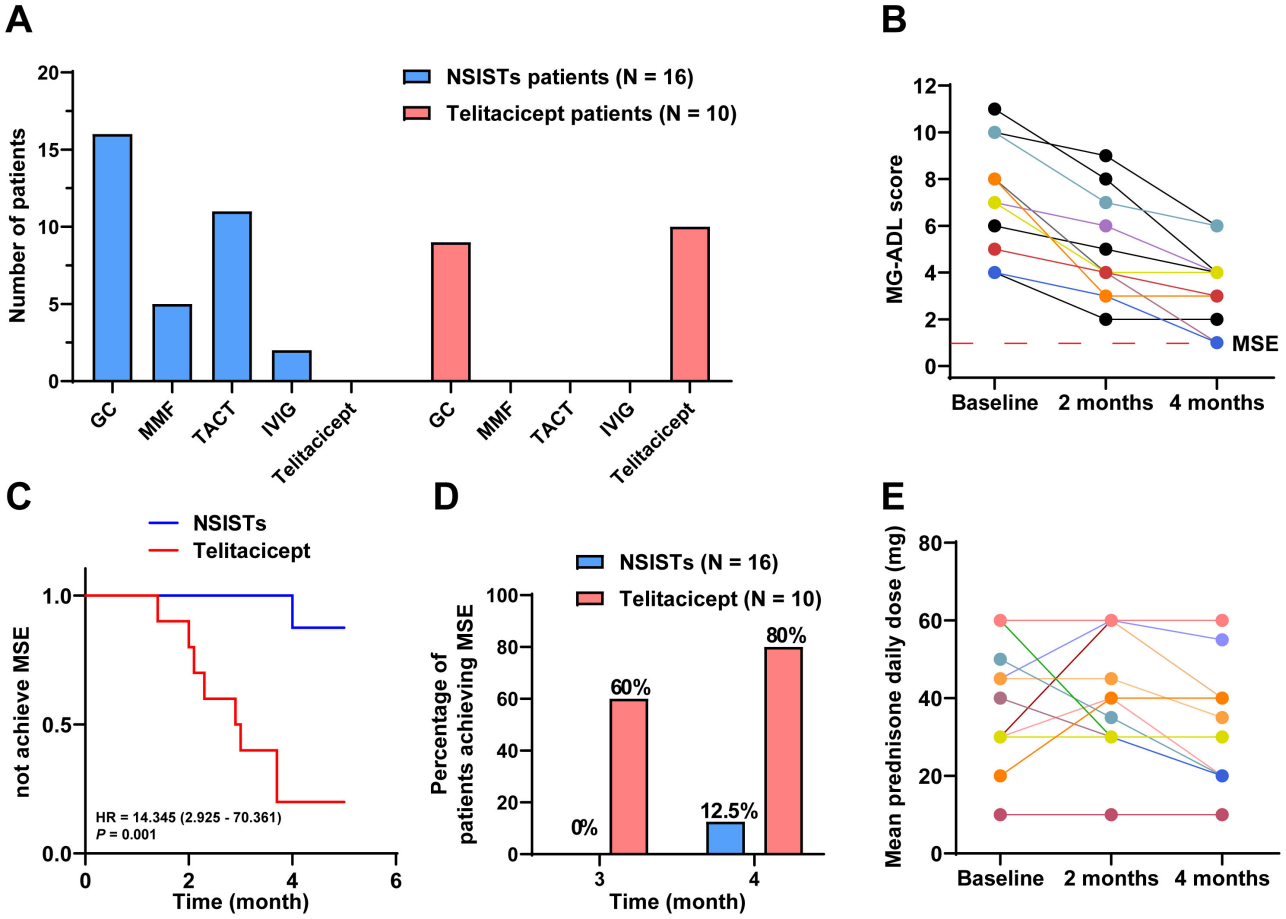
Telitacicept achieves MSE more rapidly and with greater steroid-sparing effects compared to NSISTs. **(A)** Distribution and prevalence of immunosuppressive agents and telitacicept. **(B)** Individual MG-ADL scores for NSISTs group patients at baseline, 2 months, and 4 months. **(C)** Cox regression for time to achieve MSE. **(D)** Percentage of patients achieving MSE at 3 and 4 months. **(E)** Mean daily prednisone dose for NSISTs group patients at baseline, 2 months, and 4 months.

The immunosuppressive therapy regimens used in the control group were diverse, with all patients receiving two or more immunosuppressive agents. Two patients received up to three different drugs, and six patients (37.50%) underwent thymectomy. Glucocorticoids were used in all patients (100%), followed by tacrolimus (68.75%) and mycophenolate mofetil (31.25%). IVIG was used in two patients (12.50%) (**Figure 3A**).

**Figure 3B** presents the individual MG-ADL scores of the 16 patients receiving traditional immunosuppressive therapy over the 4-month period. The Cox proportional hazards model revealed a *HR* of 14.345 (95% *CI*: 2.925-70.361) with a *P* value of 0.001, suggesting that patients in the telitacicept group were significantly more likely to achieve MSE than those in the NSISTs group. This finding highlights the superior efficacy of telitacicept in promoting earlier achievement of MSE compared to NSISTs (**Figure 3C**). Notably, 60% of the telitacicept group achieved MSE within 3 months, compared to 0% in the NSISTs group. By 4 months, 80% of the telitacicept group had reached MSE, whereas only 12.5% of the NSISTs group achieved this status (**Figure 3D**).

The average daily prednisone dosage at baseline (42.50 mg, IQR 30.00-60.00 mg), 2 months (37.50 mg, IQR 30.00-60.00 mg), and 4 months (30.00 mg, IQR 22.50-51.25 mg) for the NSISTs group is shown in **Figure 3E**. Friedman’s test indicated that there was no significant change in prednisone dosage in the NSISTs group over time (*P* = 0.091). The comparison of the average daily prednisone dosage between the NSISTs group and the telitacicept group across different time points has revealed no significant difference at baseline, with the NSISTs group having a mean rank of 12.84 and the telitacicept group a mean rank of 14.55, a *Z* score of 0.574, and a *P* value of 0.586, indicating similar levels of steroid usage at the commencement of the treatment. However, after two months of treatment, there was a marked reduction in prednisone usage in the telitacicept group, which had a mean rank of 7.45 compared to the NSISTs group’s mean rank of 17.28, with a *Z* score of -3.254 and a *P* value of 0.001, demonstrating a statistically significant decrease in steroid usage. By the fourth month, the telitacicept group continued to exhibit a lower steroid usage, with a mean rank of 7.25, in contrast to the NSISTs group’s mean rank of 17.41, a *Z* score of -3.323, and a *P* value of < 0.001, further confirming the significant effect of the telitacicept group in reducing steroid usage, as illustrated in the **Table 4**. These findings suggest that the telitacicept was more effective in sparing steroids compared to traditional immunosuppressive therapies.

**Table 4:** Comparison of the average daily prednisone dosage at baseline, 2 months, and 4 months between the telitacicept and control (NSISTs) group.

Time	NSISTs	Telitacicept	*Z* score	*P* value
	Median (IQR)	Median (IQR)		
baseline	42.50 (30.00, 60.00)	45.00(30.00, 67.50)	0.574	0.586
2 months	37.50 (30.00, 60.00)	15.00 (3.75, 30.00)	-3.254	0.001
4 months	30.00 (22.50, 51.25)	6.25 (0.00, 18.75)	-3.323	< 0.001

SD, Standard Deviation; IQR, Interquartile Range.

## 4 Discussion

This study aims to evaluate the clinical efficacy of telitacicept as an alternative to NSISTs in the treatment of MG, as well as its potential in reducing steroid use. The results demonstrate that telitacicept has a significant advantage in rapidly inducing MSE and shows promising clinical value in reducing steroid usage.

According to international consensus guidelines, the therapeutic goal for MG is to achieve a minimal manifestation state (MMS) or better (in the MMS state, MG is asymptomatic or functionally impaired, with only mild weakness in certain muscle tests), with side effects not exceeding grade 1 (23). A key goal in the treatment of MG is to induce and maintain MMS early (24, 25). However, the definition of MMS is somewhat ambiguous, and its assessment can be challenging due to the lack of objective evaluation criteria (5). In contrast, MSE is more operationally feasible. Given that our study was retrospective in nature, we chose to use MSE as the primary endpoint due to its greater practicality and ease of assessment.

Although combining non-steroidal immunosuppressive drugs may help reduce steroid use, their non-specific actions generally do not fully relieve symptoms and are associated with various side effects, such as infections and malignancies (12–15). Clinically, approximately 15% of gMG patients still experience treatment intolerance or poor symptom control, known as refractory MG, even with the use of steroids in combination with traditional immunosuppressants (15, 26). Hence, there is an urgent clinical need for a medication that can rapidly and effectively control the disease while reducing long-term steroid usage.

B cells play a pivotal role in many autoimmune diseases, including SLE, Rheumatoid Arthritis (RA), and MG. Currently, B cell-targeted therapies, such as rituximab (a monoclonal antibody against CD20-positive B cells) and belimumab (a monoclonal antibody against B cell-activating factor, BAFF), are clinically used (17, 27, 28). However, the efficacy of rituximab and belimumab is limited due to the central role of plasma cells in antibody production and the involvement of APRIL in regulating plasmablast proliferation and immunoglobulin class switching by augmenting the survival and differentiation of B cells (18, 29–33). Telitacicept, as a novel dual inhibitor of BAFF and APRIL, shows promise in the treatment of autoimmune diseases by affecting both B cells and plasma cells, thereby modulating antibody production (12). Additionally, since the TACI receptor is also present on T cells, telitacicept inhibits T cell activation (18). Therefore, telitacicept offers a new direction for the treatment of autoimmune diseases like MG.

In a study involving 85 patients with acetylcholine receptor antibody-positive gMG, the timing and incidence of achieving MSE were investigated over a 3-year follow-up period after the initiation of immunotherapy. The results indicated that MSE was achieved in 37.6%, 45.2%, 55.8%, 60.3%, and 57.1% of patients at 3, 6, 12, 24, and 36 months post-treatment, respectively (5). In contrast, our study found that 80% of MG patients treated with telitacicept successfully reached MSE within the first 4 months, highlighting telitacicept’s potential in rapidly inducing MSE in patients. Furthermore, compared to NSISTs, the telitacicept treatment group had a significantly higher proportion of patients reaching MSE within 3 months (60% vs 0%), with this proportion increasing to 80% at 4 months, whereas only 12.5% of patients in the NSISTs group achieved MSE. Cox regression analysis also revealed that the proportion of patients not reaching MSE was significantly higher in the NSISTs group than in the telitacicept group (*HR* = 14.345, 95% *CI*: 2.925-70.361, *P* = 0.001), indicating that telitacicept is more effective than NSISTs in controlling MG symptoms in the short term and in rapidly inducing MSE, further supporting its potential as an alternative to NSISTs.

A significant therapeutic goal in MG is to minimize the use of corticosteroids while maintaining symptom control (34). In terms of steroid-sparing effects, the results of telitacicept are equally remarkable. According to the Friedman test, the mean daily dose of prednisone significantly decreased in the telitacicept treatment group during the study period (*P* < 0.001). This finding is consistent with previous studies on telitacicept (18, 19), indicating that it can effectively reduce the use of prednisone and mitigate the side effects of long-term steroid therapy. In MG patients, long-term steroid use can lead to adverse reactions such as osteoporosis and diabetes, making steroid-sparing treatment of great clinical significance (13, 15, 35). Further analysis showed significant differences in prednisone dosage between the telitacicept and NSISTs groups at 2 and 4 months (*P* = 0.001 and *P* < 0.001, respectively), suggesting that telitacicept is more effective than traditional immunosuppressive therapies in sparing steroids. This finding emphasizes the potential value of telitacicept in reducing MG patients’ dependence on steroids, potentially positioning it as an independent alternative to NSISTs and opening new avenues for patient treatment.

Efgartigimod, as an FcRn receptor antagonist, rapidly improves disease conditions by competing with endogenous IgG for FcRn binding sites, preventing IgG recycling and promoting its degradation in lysosomes (18). However, it has a short half-life of only 3-5 days and needs to be administered weekly. Studies have shown that the efficacy of repeated cycles is similar, with rapid disease relapse after each cycle and almost complete loss of efficacy after a 4-week withdrawal (13, 36–38). This is likely due to its short half-life and inability to inhibit upstream B cells and antibody production, which can cause fluctuations in disease control if used for maintenance therapy. Telitacicept, on the other hand, by suppressing B cell differentiation, development, and antibody production upstream in the immune mechanism, offers a longer maintenance period compared to efgartigimod. Therefore, it is more suitable for a lasting response. In our study, we observed that 6 MG patients who received sequential treatment with efgartigimod and telitacicept showed stable disease status, with no significant increase in MG-ADL scores after treatment. This suggests that telitacicept may play an important role in stabilizing MG patients’ conditions. As a B cell-targeted therapy, telitacicept reduces the number of long-lived plasma cells, thereby decreasing the production of autoantibodies, which could be a key mechanism in stabilizing MG disease during sequential treatment (17). Additionally, compared to the 4-week treatment with efgartigimod, patients had a significantly lower average daily dose of prednisone after 12 weeks of treatment with telitacicept (*P* = 0.005), further confirming its effectiveness as a steroid-sparing therapy.

However, there are certain limitations in this study. Firstly, the sample size is relatively small, which is primarily attributed to the off-label use of telitacicept. Additionally, as a retrospective analysis, the study may be subject to inherent selection and observation biases. Secondly, the study primarily focuses on short-term treatment outcomes and lacks long-term follow-up data. Moreover, ocular myasthenia gravis (OMG) is generally considered a milder subtype, and research has shown that about 79.8% of OMG patients can achieve MMS or better clinical status, with a median time to MMS of 4 months (39). The relatively high proportion of OMG patients among the 10 MG patients treated with telitacicept in this study could potentially affect the generalizability of the overall results. This is because OMG patients typically have a better prognosis and may achieve MSE more readily in the short term, which could lead to an overestimation of telitacicept’s effectiveness in MG. Furthermore, we noted that 6 of the 10 patients who received telitacicept had previously been treated with traditional immunosuppressive agents, which may have influenced the therapeutic effect of telitacicept due to possible drug interactions or residual drug concentrations.

To address the limitations of current research, there is an urgent need to conduct large-scale, prospective, randomized controlled trials to validate the efficacy and safety of telitacicept in MG patients, and to assess its long-term effects. To achieve this, we have initiated a prospective study, which is registered on ClinicalTrials.gov (NCT06723548). This study will enroll more patients to increase the sample size and explore differences in treatment responses among different clinical subtypes of MG. This approach will further validate our findings and enhance the reliability and generalizability of the results.

In conclusion, telitacicept, as a novel immunotherapeutic agent, offers an effective alternative to traditional immunosuppressive treatments for MG patients. Its rapid clinical improvement and significant steroid-sparing effects, particularly in reducing the side effects of long-term steroid therapy, are of great clinical significance. Future research should further explore its potential applications in various immune-mediated diseases and assess its long-term efficacy and safety.

## Data Availability

The raw data supporting the conclusions of this article will be made available by the authors, without undue reservation.
