# Supplementary material for: Telitacicept as an alternative to non-steroidal immunosuppressive therapies in the treatment of myasthenia gravis: a study on clinical efficacy and steroid-sparing effect
Source: Front Immunol. 2025 Mar 24;16:1549034. doi: 10.3389/fimmu.2025.1549034 (PMC11973063; doi:10.3389/fimmu.2025.1549034)
Supplement: Supplementary file 1 [file Table1.docx]

**Supplementary Table 1. Baseline Characteristics of Patients Treated with Traditional NSISTs**

| **Patient No.** | **Gender** | **Age (years)** | **Disease duration (months)** | **MGFA class** | **Thymus status** | **Thymectomy** | **Ab status** | **RNS** | **Fatigue test** | **Comorbidities** | **Baseline MG-ADL Score^a^** | **Initial dose of GCS (mg/d)** |
| --- | --- | --- | --- | --- | --- | --- | --- | --- | --- | --- | --- | --- |
| 1 | Female | 63 | 50 | II | Thymoma | Y | NA | NA | Positive | HTN, DM | 5 | 60 |
| 2 | Female | 32 | 12 | II | Normal | N | MuSK-Ab | Negative | Positive | N | 8 | 20 |
| 3 | Female | 57 | 10 | II | Thymoma | Y | AChR-Ab | Negative | Positive | N | 7 | 30 |
| 4 | Female | 24 | 2 | I | Normal | N | NA | Positive | Positive | N | 4 | 60 |
| 5 | Female | 49 | 12 | II | Normal | N | AChR-Ab | Negative | Positive | N | 7 | 30 |
| 6 | Female | 21 | 120 | III | Thymic hyperplasia | Y | AChR-Ab | NA | Positive | Hypercholesterolemia | 8 | 60 |
| 7 | Female | 58 | 12 | I | Normal | N | NA | Positive | Positive | Hepatic dysfunction, Chronic gastritis | 5 | 30 |
| 8 | Female | 59 | 4 | II | Normal | N | MuSK-Ab | Positive | Positive | HTN, AS, Lacunar infarction | 4 | 40 |
| 9 | Female | 71 | 2 | III | Normal | N | AChR-Ab + Tintin-Ab | Positive | Positive | HTN, DM, Hypothyroidism | 10 | 60 |
| 10 | Female | 53 | 3 | II | Normal | N | AChR-Ab | Positive | Positive | Uterine fibroids | 4 | 50 |
| 11 | Female | 65 | 27 | III | Normal | N | AChR-Ab | Positive | Positive | Hypothyroidism, OP | 6 | 45 |
| 12 | Female | 54 | 65 | I | Normal | N | NA | Positive | Positive | Anemia | 6 | 10 |
| 13 | Male | 59 | 1 | III | Thymoma | Y | AChR-Ab + Tintin-Ab | Positive | Positive | Left adrenal nodule, Pulmonary nodule | 11 | 45 |
| 14 | Female | 33 | 4 | III | Thymoma | Y | AChR-Ab + Tintin-Ab | NA | Positive | N | 5 | 30 |
| 15 | Female | 36 | 20 | II | Thymic hyperplasia | Y | NA | Positive | Positive | Renal calculi, Breast nodules | 5 | 60 |
| 16 | Male | 54 | 24 | III | Normal | N | Negative | Negative | Positive | TB-IGRA (+) | 10 | 30 |

Abbreviations: MGFA, Myasthenia Gravis Foundation of America; AChR-Ab, acetylcholine receptor antibodies; MuSK-Ab, muscle specific tyrosine kinase antibodies; RNS, repetitive nerve stimulation; HTN, hypertension; AS, Atherosclerosis; DM, diabetes mellitus; OP, Osteoporosis; TB-IGRA, Tuberculosis Interferon-Gamma Release Assay; MG-ADL, Myasthenia Gravis–Activities of Daily Living; Ab, antibody; GCS, Glucocorticoids; NA, not available.

^a^Total MG -ADL scores range from 0 (normal) to 24 (severe).
